# Patient reported measures of informed consent for clinical trials: A systematic review

**DOI:** 10.1371/journal.pone.0199775

**Published:** 2018-06-27

**Authors:** Katie Gillies, Alexander Duthie, Seonaidh Cotton, Marion K. Campbell

**Affiliations:** Health Services Research Unit, University of Aberdeen, Foresterhill, Aberdeen, United Kingdom; Karolinska Institutet, SWEDEN

## Abstract

**Introduction:**

The subjective assessment of the adequacy of informed consent for clinical trials, and the potential difficulties associated with it, has led several studies to develop objective measures of informed consent for clinical trials. These objective measures of informed consent are often specific to a particular population or clinical condition and largely focus on understanding of (some or all of) the key elements of informed consent. Many of the developed tools are study-specific, but some validated measures exist. Of these validated measures, those which are reported by participants are of particular interest. Whether these objective tools conceptualize and measure informed consent in the same way is not known. As such, it is not clear whether meta-analyzing data from studies reporting different tools is worthwhile. The aim of this systematic review was to critically appraise the evidence on the overall conceptualisation and item content of validated patient reported measures of informed consent for clinical trials, and to identify core domains of potential importance for informed consent.

**Methods:**

A systematic search of the literature was conducted to identify relevant articles that described the development, and/or validation, of patient-reported measures of adequacy of informed consent for randomised controlled trials. Data was synthesised by classifying the items identified into domains and sub-domains which were determined by the nomenclature reported in included studies. Both for descriptions of included studies and of the instruments reported in those studies, descriptive statistics were used to describe general information and instrument detail. A narrative synthesis of the instruments and their inter-related domains and subdomains was conducted to identify areas of both convergence and divergence.

**Results:**

The search identified 8193 citations. After screening titles and abstracts, 29 full text articles were retrieved for further assessment. Of these 29, 14 complied with our pre-specified inclusion criteria with 15 not being eligible. Of the 14 instruments, three explicitly reported a theoretical or conceptual framework underpinning their development, a further three implicitly referred to the ‘conceptual dimensions of informed consent’ or ‘principles of research ethics’ as informing their development and eight reported no guiding theoretical framework. Only three of the 14 studies reported patient or public involvement in the development of the tool. One hundred and seventy nine items were included across the 14 instruments. The primary focus of the instruments was on understanding. Five core domains were identified which included: Autonomy; Consequences; Expectations; Purpose; and Individualisation. There was substantial variability in the coverage of different domains across measures.

**Conclusions:**

This study demonstrated the variability in the theoretical underpinning, development and domain coverage of existing patient-reported measures of informed consent for clinical trials. The conceptualisation of informed consent could benefit from being extended from a narrow focus on understanding to include broader considerations of decision-making. Meaningful involvement of potential trial participants during development of measures critical for tool relevance is also lacking. The identification of the key domains relevant to all stakeholders which could be measured to assess the informed consent process for clinical trials is needed.

## Introduction

Before patients can be recruited in to a clinical trial there is both a legal and ethical requirement that they have given their informed consent to be involved [1]. This ethical and juridical requirement, enshrined in the Declaration of Helsinki and other international regulations, was established as a mechanism to protect potential participants from any undue harm from research [1, 2]. In broad terms, informed consent for research covers aspects such as capacity, disclosure, understanding, voluntariness and permission [3]. Due to the randomised nature of clinical trials and the subsequent loss of treatment choice conferred within the design, the stakes for participants are often deemed higher than other research studies and as such the regulatory requirements become further prescriptive. The requirements are such that the informed consent ‘process’ for clinical trials usually includes both verbal and written information that explains (as a minimum) the purpose and aim of the trial, research procedures, anticipated risks and benefits, end of trial provisions, source of funding, potential conflicts of interest, and researchers institutional affiliation [1]. In addition, potential trial participants should be aware of their right to refuse participation and the ability to withdraw consent at any time. For consent to be considered valid, in accordance with existing regulatory frameworks, it must be voluntary, informed and with the individual providing consent having sufficient capacity to do so [1,2]. The Declaration of Helsinki states that ‘After ensuring that the potential subject has understood the information’ the researcher should seek the participant’s freely given informed consent [1]. In addition to the current guidance, the Clinical Trials Regulation (which will come into effect within the European Union in 2019) states that for consent to be valid ‘it shall be verified that the subject has understood the information’ [4]. However, the ‘what’ and ‘how’ of ensuring or verifying that consent is valid (i.e. informed, given voluntarily and by an individual with capacity) are not specified.

The concept of ‘informed consent’ (both for treatment and for research) is often interpreted differently and operationalized inconsistently [5, 6]. There is no universally agreed measure of “good” informed consent for clinical trials that might be used to objectively evaluate whether the potential participant has understood what trial participation means for them, or indeed ensured that all other considerations appropriate to ensure informed consent has been achieved have been met. A number of trial-specific, condition-specific and more generic measures of aspects of clinical trial informed consent do exist; however, they have been noted to have variable operationalisations of informed consent and are collected through a variety of mechanisms–some reported by the participant themselves; others not [6, 7].

In a previous study, Sand et al [7] provided a useful map of the range of measures available to measure understanding in informed consent; however, there has been no investigation to date of the underlying concepts captured by different measures of informed consent (and consequently which concepts may not currently be captured by these instruments), how these may relate to each other (and more broadly to informed consent) and hence how the choice of measure might influence the results of a study investigating the quality of informed consent.

We, therefore, undertook a systematic narrative review to:

- critically appraise the evidence on the overall conceptualisation and item content of validated patient reported measures of informed consent for clinical trials; and
- identify reported core domains of potential importance for informed consent.

Recognizing that the participants’ perspective is of utmost importance when considering whether the consent process was ‘good’ or not, we focused our review only on patient-reported measures of informed consent. A consent process can fulfill all of the legal imperatives to ensure consent is informed from a process perspective i.e. provision of information, discussion, etc. However, only by asking potential participants for their perspectives can judgements be made about whether or not consent was truly informed. The findings of our review are presented in this manuscript.

## Methods

### Search methods for identification of studies

The search strategies were designed by KG in collaboration with a Senior Information Scientist to identify relevant articles that described the development, and/or validation, of patient reported measures of informed consent for randomised controlled trials (RCTs). We supplemented the MEDLINE and EMBASE strategies with the filter described by Terwee and colleagues which identifies studies on measurement properties of measurement instruments [8]. The strategies for each of the databases are presented as text in S1 Appendix. We conducted searches for relevant studies on the following pre-specified databases:

- MEDLINE (OvidSP) (1946 to 1^st^ September 2016)
- EMBASE (Ovid SP) (1980 to 2016 week 35)
- CINAHL (OvidSP) (1960 to 1^st^ September 2016)
- PsycINFO (OvidSP) (1970 to 1^st^ September 2016)

There were no language nor date restrictions. Table 1 presents number of studies identified in each database. We combined the results and removed duplicates. Reference lists of included papers were screened to identify further relevant publications.

**Table 1:** Search results for electronic databases.

Database searched	Number of hits
MEDLINE	4244
EMBASE	4001
PsycINFO	1813
CINAHL	1526
TOTAL	11871
After de-duplication	8191

#### Inclusion criteria

The purpose of this review was to identify the items included in validated measures of RCT informed consent (we did not seek to evaluate the methodological quality of these measures). However, to ensure the methodology applied was rigorous we followed the COnsensus-based Standards for the selection of health Measurement Instruments (COSMIN) Protocol for systematic reviews of measurement properties [9]. The following inclusion criteria were applied:

1. the instruments should aim to measure informed consent (or aspects of) in a clinical trial context;
2. the study sample should contain potential trial participants;
3. the study should include a questionnaire based measure (to include self-report, administered by interview; or by proxy);
4. the aim of the study should be the development of a measurement instrument and evidence of the evaluation of one or more of its measurement properties;
5. the study should be published as a full text original article;
6. articles in all languages will be assessed for eligibility.

#### Exclusion criteria

1. Studies that reported study specific measures with no details on development or validation;
2. Studies that measured informed consent for treatment or for research participation in a study other than an RCT;
3. Trials or studies evaluating the effectiveness of informed consent interventions where a questionnaire is used to measure an endpoint (without describing the development of the tool in full).

#### Selection of studies

One reviewer (KG) screened all identified titles and abstract with another reviewer (MC) screening a random 10% sample for consistency. Full text articles were assessed by one reviewer (KG) for inclusion and a random 30% sample was screened by another reviewer (MC) for consistency. Any disagreements on eligibility were discussed and consensus reached.

### Data extraction and management

Data extraction forms and tables were generated for each stage of the extraction process to standardise the information recorded and aid analysis. All data extracted and presented relates to data pertaining to the study that reported the development and validation of an instrument to measure informed consent to clinical trials. One review author (KG) independently extracted data related to study characteristics (e.g. demographics of population, host trial detail, etc) and instrument characteristics (e.g. whether theoretically informed, number of items, etc) from each included study. This was checked by a second reviewer (SC) for accuracy. General data extraction categories were split into those relating to the context of the included informed consent study and those relating to the included informed consent instrument. Specifically these were:

1. Included study characteristics: country; population; sample size; response rate; age; gender; ethnicity; employment status; and education status.
2. Included instrument characteristics: name of instrument; theoretical/conceptual framework; construct assessed; time required to complete; recall period; dimensions; patient reported outcome; response options; ease of scoring and administration; mode of administration; sample items; and patient or public involvement in development.

Data extraction and coding relating specifically to content items (i.e. individual questions) of the instrument was conducted by two reviewers (KG and AD) independently with a random sample of papers (n = 5) assessed for consistency in overlap between reviewers (by KG). Any disagreements were resolved through discussion with a third reviewer (MC) if required. Data extraction was based on the following categories: name of the tool; concept of tool (verbatim from included study); items (verbatim from included study); construct being targeted by item (verbatim from included study); domain (as defined by review authors and informed by construct targeted); and sub-domain (as defined by review authors).

### Data synthesis

The first step of data synthesis involved the coding of the items identified from the instruments of included studies into domains and sub-domains determined by the reviewers. This process followed many of the tenets of ‘directed’ content analysis [10]. Directed content analysis is guided by a structured approach that uses existing theory (or research) to identify the key concepts as initial coding categories [10]. Our initial proposed coding categories were thus informed by constructs identified from previously published regulatory guidance and existing research on informed consent. This was then supplemented by any additional constructs identified within the included manuscripts. The direct process of assigning appropriate codes to concepts discussed in manuscripts was primarily informed by consideration of how the authors reported the underlying construct. Initial allocation of codes was followed by discussion amongst two reviewers (KG and AD) to reach consensus on reviewer determined domains. This parallel coding process was carried out for 20 (11%) of the items with the remainder being coded independently by the two reviewers and results compared and discussed on completion. A third reviewer (MC) coded a random sample of 46% (n = 83) of the items to offer additional considerations and provide overall agreement on the coding categories. Items were then agreed across the main domains and sub-domains to provide an overall perspective of the conceptual framework emerging from these instruments.

Both for descriptions of included studies and of the instruments reported in those studies, descriptive statistics were used to describe general information and instrument detail. A narrative synthesis of the instruments and their inter-related domains and subdomains is presented.

## Results

**Fig 1:**
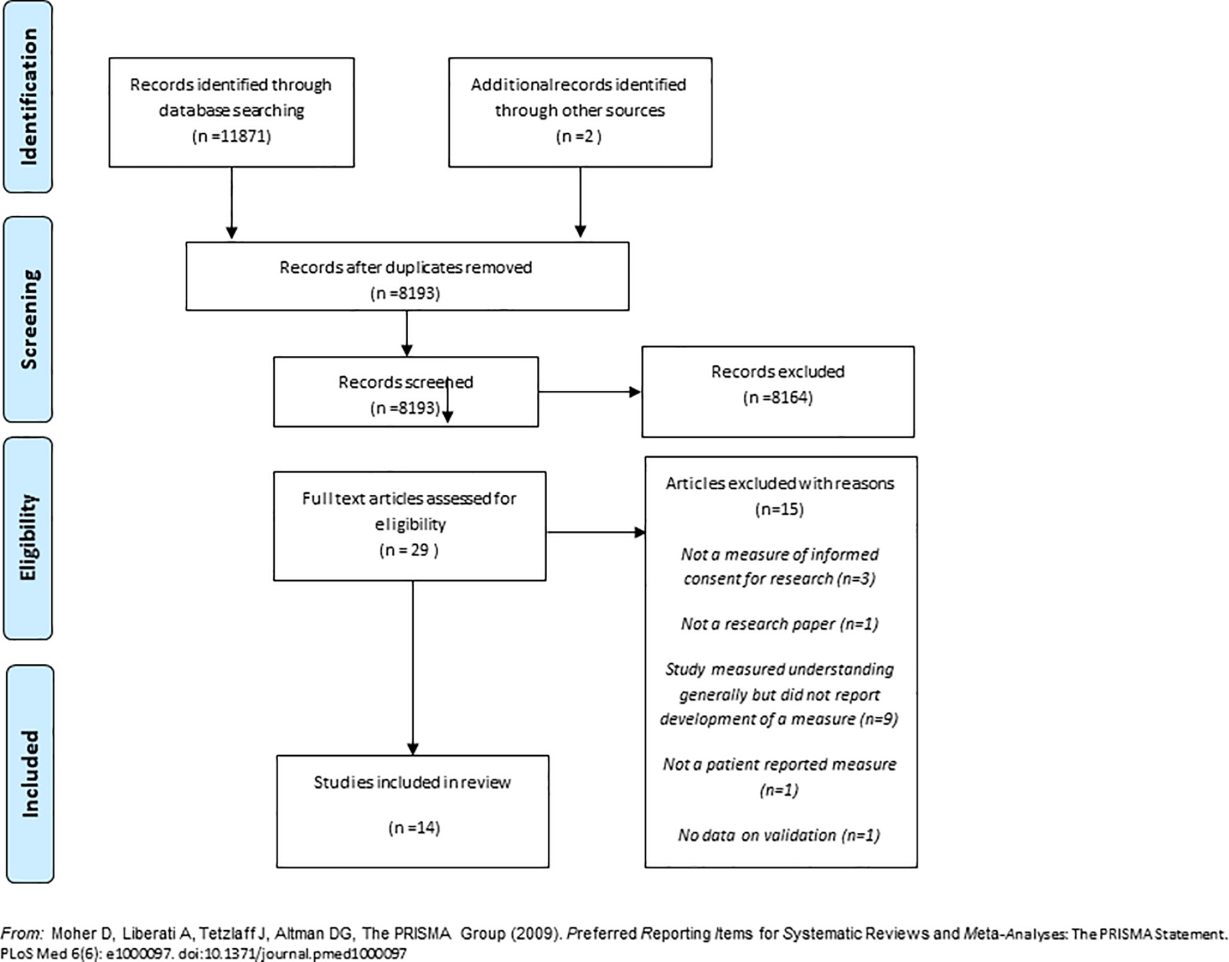
PRISMA flow diagram for identification of included studies in informed consent for RCTs patient reported measures review.

The search identified 8193 citations. After screening titles and abstracts, 29 full text articles were retrieved for further assessment. Of these 29, 14 complied with our pre-specified inclusion criteria and 15 were not eligible. Reasons for full text articles being deemed not being eligible are reported in Table 2. Full details of the search process can be seen in Fig 1.

**Table 2:** Excluded studies.

	Paper	Reason
1	Albrecht TL et al 1999	Measures patient–physician communication in cancer clinical trials. Not focussed on development of an instrument.
2	Woodward WE et al 1999	Measures level of understanding in population. Not focussed on development of an instrument
3	Marteau M et al 2001	Develop measure of informed choice in an antenatal screening context i.e. measure not developed for informed consent for RCTs.
4	Siminoff LA 2003	Not a report of primary research. Discussion.
5	Leroy T et al 2008	Measures level of understanding in population. Not focussed on development of an instrument.
6	Rounsaville DB et al 2008	Uses a quiz to measure level of understanding in population. Not focussed on development of an instrument
7	Bhansali S et al 2009	Measures level of understanding in population. Not focussed on development of an instrument
8	Frew PM et al 2010	Measure is focussed on willingness to participate in a clinical trial, not on informed consent.
9	Ballard HO et al 2011	Standard versus enhanced consent process. Not focussed on development of an instrument.
10	Behrendt C et al 2011	Measures level of understanding in population. Not focussed on development of an instrument
11	Cohn CG et al 2011	Measures informed consent but is not a PROM
12	Leroy T et al 2011	Measures level of understanding in population. Not focussed on development of an instrument.
13	Shafiq N et al 2011	Reports the questionnaire items from a study that measured levels of understanding in population. Not focussed on the development of an instrument
14	Horowitz RH et al 2013	Measures level of understanding in population. Not focussed on development of an instrument
15	Mexas et al 2014	Provides no formal analysis of test retest data to support validation of measurement properties.

### Descriptive characteristics: Included studies

The table in S1 Table summarises the basic characteristics of the included studies [11–24]. Identified studies were published between 1996 and 2013. Eleven of the 14 included studies were set within the USA, with one further study being multi-site and set across the USA, Canada, Australia and New Zealand. The two remaining studies were conducted in the United Kingdom and Italy. The clinical trial population in which the informed consent studies were set varied and ranged across: mental health trials (e.g. depression, schizophrenia); paediatric trials, oncology trials; and others. All studies included patients as participants (additionally some studies also involved nurses and others healthy volunteers). This variability was further reflected in the testing scenario sometimes being a real trial and others being a hypothetical trial. The size of the sample included in each included study also varied and ranged from nine to 1086 participants (median = 174). As expected the demographics (e.g. age, gender, ethnicity, employment and education) of participants in the included studies varied and in some cases were not reported (see S1 Table).

### Descriptive characteristics: Instruments from included studies

Information relating to characteristics of the instruments identified in the included studies is presented in Table 3. The included studies report 14 separate instruments whose aim is to measure an aspect of informed consent for RCTs. The included studies varied in their reports of what the instrument aimed to measure overall and reported concepts such as: ‘*therapeutic misconception*’; ‘*therapeutic misunderstanding*’; ‘*perceived understanding*’; ‘*patient understanding of research*’; ‘*clinical trial understanding*’; ‘*understanding information*’; ‘*comprehension of informed consent information*’; ‘*understanding and retention of trial information’*; ‘*maintaining informed consent’* (operationalised through repeated measurement*)*; ‘*quality of informed consent*’; ‘*evaluation to consent’* (relates to evaluating capacity); ‘*autonomous authorisation’*; ‘*assessing decisional capacity’*; and ‘*decision making’*. Four of the included studies reported development of an instrument to assess competence, or capacity, of research subjects to consent to participation in RCTs i.e. the studies were set amongst participants who may have diminished decisional capacity e.g. early stage Alzheimer’s, schizophrenia. [11,16, 23, 24].

**Table 3:** Characteristics of included instruments.

	Instrument	Theoretical/ conceptual framework	Construct assessed	Time required to complete (mins)		Recall period	Dimensions (number of items)	Response options (range)	Ease of scoring and administration (range of scores)	Mode of administration (i.e. self-completion)	Sample items	PPI
1.	MacArthur Competence Assessment Tool–Clinical Research (MacCAT-CR) Applebaum et al 1999*	None reported	1. Understanding (U) 2. Appreciation (A) 3. Reasoning (R) 4. Ability to communicate a choice (C)	15–20		Measured at 2 weeks then again at 8–10 weeks.	Total = 21 items • Understanding (n = 13) • Appreciation (n = 3) • Reasoning (n = 5) (from Resnick et al 2007)	Open-ended	Answers rated 2, 1, or 0 on the basis of objective criteria.	Trained interviewers verbally administered the instrument.	What is the purpose of the research project I described to you? (U) Do you believe that you have been asked to be in this study primarily for your benefit? (A) What is it that makes [the subject’s preferred option] seem better than [the nonchosen options]? (R)	None reported.
2.	Therapeutic Misconception (TM) *Applebaum et al 2012*	Theoretical framework. Specifically based on 2 dimensions: unreasonable beliefs, based on misunderstandings of the methods of the research based on (1) individualisation of the intervention being delivered and (2) benefits from participation. Incorporated a third dimension, on the purpose of the research to benefit future patients.	1. Individualisation 2. Benefit 3. Purpose	45 mins to complete study procedures Note: The time required to complete was only reported for the development stage which involved a larger set of items (n = 28) completed and followed up with a semi-structured interview. Likely completion time for final 10 items instrument is less than 45 minutes but was not reported in paper.		Up to 2 months	Total = 10 items • Individualisation (n = 3) • Benefit (n = 3) • Purpose (n = 4) Each at different levels: • General research • Specific project • Participants treatment	Data was collected using a mixed-methods approach. Quantitative data was collected using a closed item questionnaire with responses on a 5 option likert scale. Qualitative data was collected in interviews using structured open-ended questions	Quantitative: frequencies by category: agree; mostly agree; neither agree nor disagree; mostly disagree; disagree. Qualitative interviews coded using the 3 pre-determined codes for TM–I, B, P.	Trained interviewers verbally administered the instrument over the telephone.	People in this study may not do as well as they would in usual treatment (B) The main purpose of the study is to help people in the future, whether or not it helps me(P) Researchers always try to provide each person in a study the treatment that best meets that persons individual needs (I)	None reported apart from in pilot
3	Therapeutic Misunderstanding Scale (TMU) *Chou & O’Rourke 2012*	Informed by a three-facet conceptual model that links to theories of therapeutic misunderstanding.	1. Therapeutic misconception (TM) 2. Therapeutic misestimation (TE) 3. Therapeutic optimism (TO)	No data		No data	Total = 20 items • Therapeutic misconception (n = 9) • Therapeutic misestimation (n = 6) • Therapeutic optimism (n = 5)	5 point Likert type format	Quantitative: frequencies by category: strongly agree; agree; don’t know; disagree; strongly disagree.	Self-completion online	The main reason that people will be recruited for this study is so that they can benefit from the special treatment in this research project (TM) The treatment I receive in this clinical trial would cure my illness (TE) There are many ways my participation in this study would help me (TO)	None reported
4	Process and Quality of Informed Consent (P-QIC) *Cohn et al 2012*	None reported	1. Information (I) 2. Communication (C)	No data		No data. Implicit that measured at point of consent	Total = 20 items • Information (n = 14) • Communication (n = 6)	5 point Likert type format	Quantitative: frequencies by category: done well; done; done poorly; not done; not applicable.	Observer completed	Greets and shown interest in the participant as a person (C) Provides step-by step information about the study and procedures (I)	None reported
5	Informed Consent Questionnaire(ICQ) *Guarino et al2006*	None reported	1. Perceived understanding (U) 2. Satisfaction (S) 3. Voluntariness of consent (V)	No data		No data. Implicit that measured at point of consent	Total– 10 items • Perceived understanding (n = 6) • Satisfaction (n = 3) • Voluntariness of consent (n = 1) 1 open ended question for comments	Q1-4: Dichotomous Q5-10: 4 Point Likert type format	Q1-4: Yes/No Q5-8: Categories: not at all; somewhat; mostly; yes, completely.	Self-completion	Do you remember signing a document that indicated that you understood your rights as a research subject and that you were willing to participate in this study? (U) Would you participate as a research subject if this study were repeated? (S) Did participating in this study meet your expectations? (V)	Yes Developed by study investigators and then reviewed and modified by a group of 5 patients.
6	Questionnaire-Patient Understanding of Research (Q-PUR) *Hutchison et al 2007*	None reported	Not explicit but suggests largely measures understanding of: 1. Randomisation 2. Intervention and comparator 3. Which treatment is best 4. Value of standard treatment	Patient: Previous RCT	8.8 (range 5–20)	No data N/A	Total = 13 items	Four part options	Multiple-choice test response, always with one option of ‘don’t know’.	Self-completion	Research with patients is carried out….. If you do not want to take part in a trial….	Yes 4 patient involved in design and a further 2 in refinement.
				Patient: No RCT	10.6 (range 5–20)							
				Research Nurse	8.8 (range 5–20)							
7	University of California, San Diego Brief Assessment of Capacity to Consent (UBACC) Jeste et al 2007*	None reported	5. Understanding (U) 6. Appreciation (A) 7. Reasoning (R)	<5		No data	Total = 10 items • Understanding (n = 4) • Appreciation (n = 5) • Reasoning (n = 1)	Mixture of open-ended questions and closed ended questions	Answers scored according to code guidebook. Each answer scored on a scale of 0–2, where 0 is clearly incapable, 1 is partially appropriate and 2 is clearly capable. Total score of 20.	Verbally administered by researcher.	Do you believe this is primarily research of primarily treatment? (U) Do you have to be in this study if you do not want to participate? (A) What makes you want to consider participating in this study? (R)	Experts, which did not include patients, were consulted to ensure content validity. An advisory groups, which did include patients, consulted across project.
8	Quality of Informed Consent (QuIC) *Joffe et al 2007*	Conceptual framework considered: existing theoretical work on therapeutic misconception; regulations governing research; recommendations of the National Cancer Institute’s working group.	1. Objective understanding 2. Subjective understanding Based on 13 domains identified from regulatory documents on informed consent.	7.2 (range 2.5–12.8)		No data. N/A	Total = 34 items • Part A: Objective understanding (n = 20) • Part B: Subjective understanding (n = 14)	Part A: 3 point Likert type format Part B: 5 point Likert type format	Part A categories cover; disagree, unsure, agree. Part B categories cover: a 1–5 scale with ‘I didnt understand this at all ‘anchored to 1 and ‘I understood this very well’ anchored to 5.	Self-completion	I have been informed how long my participation in this clinical trial is likely to last (A) The fact that your treatment involves research (B)	None reported apart from pilot
	Deaconess Informed Consent Comprehension Test (DICCT) *Miller C et al 1996*	None reported	Comprehension (of information contained in informed consent documents)	Mean = 7		Approx.1 hour post-consent	Total = 14 items	Open ended questions	Responses are scored as: 2 points for a correct answer; 1 point for a correct but incomplete answer or demonstrating poverty of content; and 0 for incorrect or no answer. Total possible score of 28.	Study investigator read questions aloud and answers are given verbally and transcribed verbatim.	What is the purpose of the study? Your participation in this study is entirely voluntary. What will happen if you refuse to be in the study?	None reported
9	Index of Clinical Trial Understanding (ICTU) *Miller J et a 2011l*	None reported	Knowledge of key clinical trial domains: control group; randomisation; standard therapy; placebo; and trial purpose.	Less than 10 mins.		Not reported	Total = 7 items	Mixture of open-ended, multiple choice and likert type formats.	Open-ended responses coded using a coding guide and given a score of 2 for correct answers, multiple choice questions were scored based on correct answers and given a score of 1, and likert items ranged from 0 (completely disagree) to 10 (completely agree) And asked for participants level of agreement with correct answers given a score of 1. Total possible score of 9.	No explicit report, implicitly suggests self-completion.	If you were read a story about a clinical trial in a newspaper, would you have a clear understanding of what is involved in a clinical trial, a general sense about clinical trials, or not much idea? Clinical trials are essential to advancing medical science and improving the standard of care. (completely agree-completely disagree)	None reported.
10	Decision Making Control Instrument (DMCI) *Miller V et al 2011*	None reported.	Voluntariness, locus of control, self-efficacy, and decision making.	Not reported		Not explicit. Approached as soon as possible following index decision.	Total = 28 items in the initial experimental tool, scaled down to a core of 9 items	6 point Likert type formats	Categories covered: strongly disagree; disagree; somewhat disagree; somewhat agree; agree; strongly disagree.	Self-completion.	I made this decision. I was powerless in the face of this decision. Someone took this decision away from me.	Focus groups and interviews with patients to determine item inclusion.
11	Porteri Study 2007*	None reported	Understanding of information about research protocol.	5–8 mins		Completed as soon as possible following index decision.	Total = 12 items	Open and closed questions.	Open questions coded using guided answers. Closed questions were multiple choice, each with 3 possible answers. Total possible score of 24.	Verbally administered by researcher with participants able to read alongside.	What is the aim of the study? Please, list the medical examinations which you will undergo for the aim of this study.	None reported
12	Assessment of Sustained Informed Consent (ASIC) *Prentice et al 2007*	No theoretical framework reported. Informed by conceptual dimensions of informed consent.	Knowledge and understanding with particular reference to sustained consent over time.	No data		No data	Total = 7 items	Open ended questions	Answers dichotomised to pass/fail and scored as 0 or 1. Total possible score of 7.	Verbally administered by researcher.	Are you required to participate in this research study? Are you allowed to withdraw from the study?	None reported
13	Evaluation to Sign Consent (ESC) Resnick et al 2007*	No explicit theory reported.	Knowledge and understanding in relation to potential risks; randomisation; expectations of them as participants; what to do if unhappy (i.e. discomfort or withdrawal).	No data		Completed before consent for the parent trial	Total = 5 items	Open ended questions	Answers were scored as correct based on a pre-determined list.	Verbally administered by research evaluators.	What are two potential risks? What is expected from you, the resident?	None reported
14	Brief Informed Consent Evaluation protocol (BICEP) *Sugarman et al 2005*	No explicit theory reported. Conceptual dimensions: therapeutic misunderstanding; voluntariness; and understanding.	Autonomous authorisation	7.7 min(2.9)		Immediately following consent to the parent trial	Total = 15 items • Informed Consent Aggregate Acores(ICAS) (n = 10) • Therapeutic Misconception Aggregate Score (TMAS) (n = 5)	Closed-ended or coded	Items scores 1 for yes and 0 for no. Total score of 15.	Verbally administered by researcher over the telephone.	What is the primary purpose of [parent study]? What are the benefits to you for participating in the [parent study]?	None reported

*Developed to assess competence of research subjects to consent to participation in RCTs

Of the 14 instruments, three explicitly reported a theoretical or conceptual framework underpinning their development (theories of therapeutic misunderstandings and or misconception [12, 13, 17]): a further two [22, 24] implicitly referred to the ‘conceptual dimensions of informed consent’ or ‘principles of research ethics’ as informing their development and nine reported no guiding theoretical framework. Linked to this, some instruments were explicit with regard to which constructs they were measuring while others were more vague.

The time required to complete the measure ranged from less than 5 to 20 minutes (median 8.4 mins). Five studies did not report time required to complete [13, 14, 20, 22, 23] and one reported completion time as ‘study procedures’ that included additional procedures as well as instrument completion [12]. Half of the included studies did not report the recall period assessed using the instrument with the seven remaining studies varying from point of consent to 10 weeks post consent.

The number of items per instrument varied across the 14 measures and ranged from 5 to 34 (median = 14) with a cumulative total of 179 individual items. There was some level of duplication or overlap across the 179 items. Specifically, 156 (87%) measured understanding or knowledge of domains, nine items (5%) measured decisional control (and focused on perceived voluntariness with all items identified from one tool [20]), eight items (4%) measured appreciation (of aspects such as expectations and consequences of participation) and six items (3%) measured reasoning. All of the appreciation and reasoning items are attributable to two tools that both aimed to measure patient’s competence or capacity to consent for research [11, 16]. Response options of instruments also varied with some being open-ended responses and others being Likert type questions. Mode of administration also varied with the majority being self-completed but others being administered by trained interviewers either face-to-face or over the telephone.

Finally, five of the 14 studies reported patient or public involvement during some stage in the development of the tool [12, 14, 15, 17, 20]. Two studies directly involved patients in identifying core content for inclusion in the tool [15, 20]; one study involved patients in reviewing and modifying the tool before piloting [14]; and two studies worked with patients during the piloting of the tool [12, 17].

### Item domain and sub-domain classification

As mentioned above, the majority of items contained across all measures assess participants understanding in relation to specific features of the trials. Other aspects of cognition reported to be measured related to decisional control, reasoning, and appreciation. To identify which aspects of understanding were being assessed, we coded individual items into domains and sub-domains that broadly captured the underlying construct being measured (as described in the Methods section). Table 4 presents a summary of the item classification, including definitions (with example items), according to coded domains and subdomains. S1 Text provides coding of all items across the included instruments.

**Table 4:** Domains and subdomains identified across individual items of measures identified in the review.

Domain (N (%))	Definition	Subdomain (N (%)) *Content relates to understanding of specific sub domain*.
Autonomy 26 (15)	A potential participant’s right to make choices, to hold views, and to take actions based on personal values and beliefs. *e*.*g*. *Are you required to participate in this research study?*	Motivation—1 (4) Process—1 (4) Voluntariness—24 (92)
Consequences 62 (35)	Any event that occurs as a result of participating in the trial and are contingent on participation. Consequences can be reinforcers (i.e. increase participation) or punishers (i.e. decrease participation). *e*.*g*. *Do you feel that the potential benefits of participation in this study were explained?*	Alternatives—4 (6) Benefit—29 (46) *Benefit/Risk—5 (8) Confidentiality- 4 (6) Disadvantage/Risk- 13 (21) Process -3 (5) Voluntariness -5 (8)
Expectations 17 (9)	Beliefs about what will (or did) happen when participating in the trial. Tends to focus on the future. Can give rise to disappointment if a less than favourable outcome occurs. *e*.*g*. *Were the treatments more difficult than you expected?*	Experience—3 (18) Positive Beliefs -2 (12) Process—8 (47) Satisfaction—4 (23)
Purpose 66 (37)	The specific aim or process requirements of the study. *e*.*g*. *Were the treatments more difficult than you expected?*	Aim -8 (12) Alternatives- 1 (1) Benefit- 6 (9) *Benefit/Risk—9 (14) Confidentiality—1 (1) Process—13 (20) Randomisation - 15 (23) Therapeutic misconception—10 (16) Uncertainty—2 (3) Voluntariness– 1 (1)
Individualisation 8 (4)	The belief that treatment choices will be individualised for the potential trial participants specific needs. *e*.*g*. *The treatment /intervention I would receive in this study will be adapted according to my needs*, *like the treatment from any other doctor*.	Process– 1 (12.5) Therapeutic Misconception—6 (75) Voluntariness—1 (12.5)

*Benefit/Risk: These items were assessing understanding of both benefit and risk information together

**Fig 2:**
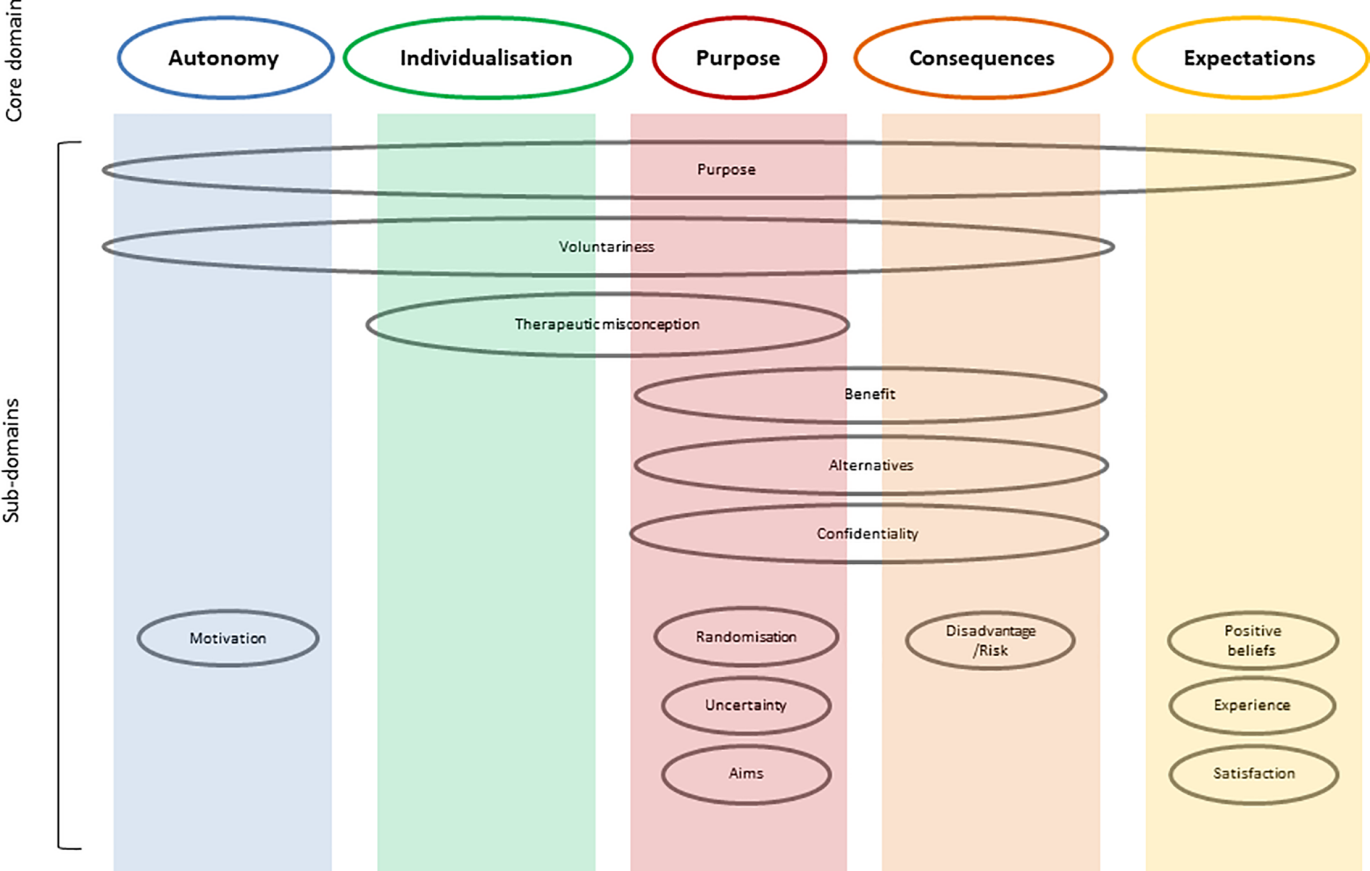
Conceptual diagram of domains from RCT informed consent validated measures.

Our review identified five core domains across the 179 items (which spanned the core cognitive concepts of understanding, decisional control, appreciation, and reasoning). These core domains were constructed as: Autonomy; Consequences; Expectations; Purpose; and Individualisation. These domains are conceptually distinct but some are more intimately connected than others e.g. consequences and expectations. Consequences (n = 62) and purpose (n = 66) were the most commonly identified domains and individualisation (n = 8) the least. These core domains could be further categorised into discrete subdomains. Including: aim (of the trial to which they were consenting); alternatives; benefit; confidentiality; disadvantage or risk (of interventions); experience; motivations; positive beliefs; process; randomisation; satisfaction; therapeutic misconception; uncertainty; and voluntariness. The most commonly identified subdomain was ‘benefit’ (n = 35/20%) and ‘motivations’ the least (n = 1 / 0.5%). It is important to note that subdomains were not exclusively linked to one core domain. For example, ‘process’ was associated with all domains whereas ‘confidentiality’ was only linked to ‘consequences’ and ‘purpose’. Fig 2 provides an overview of the relational intersections of sub-domains across core domains identified in patient reported measures of informed consent for clinical trials.

Table 5 provides a summary of domain content identified in the informed consent measures included in our review. Fourteen of the included instruments could be classed as being multi-dimensional based on their coded domains. The Index of Clinical Trial Understanding (ICTU) was the only measure that could be considered unidimensional due to it exclusively including items that could be coded to one domain: purpose [19]. However, within the purpose domain, the ICTU patient reported measure did capture aspects relating to aim, process and randomisation. Across the 14 patient reported measures, only 1 included all 5 identified domains: the MacArthur Competence Assessment Tool–Clinical Research (MacCAT-CR) [11]. The MacCAT-CR was developed to assess depressed patients capacities to consent to research and covers cognitive aspects of appreciation and reasoning in addition to understanding [11].

**Table 5:** Inclusion of core domains across included measures ranked by frequency of domains.

*Instrument*	Autonomy	Consequences	Expectations	Purpose	Individualisation	# of domains
MacArthur Competence Assessment Tool–Clinical Research (MacCAT-CR)	**+**	**+**	**+**	**+**	**+**	**5**
Quality of Informed Consent (QuIC)	**+**	**+**	**+**	**+**	**-**	**4**
University of California, San Diego Brief Assessment of Capacity to Consent (UBACC)	**+**	**+**	**+**	**+**	**-**	**4**
Deaconess Informed Consent Comprehension Test (DICCT)	**+**	**+**	**+**	**+**	**-**	**4**
Evaluation to Sign Consent (ESC)	**+**	**+**	**+**	**+**	**-**	**4**
Brief Informed Consent Evaluation protocol (BICEP)	**+**	**+**	**+**	**+**	**-**	**4**
Therapeutic Misunderstanding Scale (TMU)	**-**	**+**	**+**	**+**	**+**	**4**
Therapeutic Misconception (TM)	**-**	**+**	**-**	**+**	**+**	**3**
Informed Consent Questionnaire(ICQ)	**-**	**+**	**+**	**+**	**-**	**3**
Questionnaire-Patient Understanding of Research (Q-PUR)	**+**	**+**	**-**	**+**	**-**	**3**
Porteri et al (2007)	**+**	**+**	**-**	**+**	**-**	**3**
Assessment of Sustained Informed Consent (ASIC)	**+**	**-**	**+**	**+**	**-**	**3**
Decision Making Control Instrument (DMCI)	**+**	**-**	**-**	**-**	**+**	**2**
Index of Clinical Trial Understanding (ICTU)	**-**	**-**	**-**	**+**	**-**	**1**
*Total count across tools*	***10***	***11***	***9***	***13***	***4***	

## Discussion

This review of patient reported measures for informed consent for clinical trials is, to our knowledge, one of the first to systematically characterise the item content across these measures into individual outcome domains highlighting the heterogeneity that exists across outcome measures reporting to capture the same outcome. Importantly, this review further underlines the predominant lack of input from potential trial participants during development to aid in identification of what aspects of the informed consent process for trials matters to participants and should therefore be considered important for measurement when assessing the process–a perfect contradiction.

We identified 179 individual items across 14 instruments, the majority of which (87%) assessed understanding, and the items of which could be conceptualised into 5 core domains: Autonomy; Consequences; Expectations; Purpose; and Individualisation. A range of discrete subdomains were identified across these core domains relating to content, such as: aim; benefit; disadvantage or risk; satisfaction; therapeutic misconception. The majority of instruments were multidimensional although the coverage of domains was highly variable with most measures only addressing a subset of domains–with one measure (ICTU) being unidimensional in its content. This variability in coverage and focus of the measures make any future wish to synthesise outcome data from across these measures problematic, suggesting the need for the development of a core outcome set to inform the domains that all future studies should report. This work is now in development by the authors [25].

Our review showed that the majority of instruments lacked a theoretical framework to inform their development. This calls into question their construct validity (i.e. the extent to which the instrument tests the theory it is measuring) and raises concerns about their ability to accurately measure the underlying concept of informed consent. In other words, our review may have failed to identify domains and sub-domains of relevance for informed consent to clinical trials due to the lack of key content included in the instrument development. Measures that did report the use of theory to inform their development also varied in their conceptualisation of informed consent and as such associated domains and sub-domains relating to constructs varied. Thus providing further evidence that the over-arching concept (in this case ‘informed consent to trials’) which they aim to measure is likely not the same between measures.

Whilst all included instruments were patient–reported, only three included trial participants or patients in the development phase pre-pilot. This lack of stakeholder input also raises questions about content validity and specifically whether these measures represent items that are of importance to potential trial participants [25].

Our review further highlighted the dominant focus of measures on the “understanding” of trial specific information–this concentration on understanding has also been raised by a number of other commentators [6,7, 26]. Whilst understanding of information is an important component of clinical trials informed consent, many of the existing measures lack consideration of other aspects that might be important for the decision making process (e.g. preference construction, affective forecasting and integration of information with personal values and goals) [27, 28]. Only a few studies have measured and reported specific decision making outcomes (such as decision conflict, decision regret and deliberation) during the informed consent process [29–34]. Interventions that aim to provide a more holistic approach to informed consent and go beyond solely seeking to improve understanding of trial information (by considering aspects of decision making that could also be important) are needed [28, 32, 33, 35]. As these decision-focused, and other, interventions to support the informed consent process for clinical trials become more widespread, it is likely that the instruments used to evaluate their effectiveness will also progress. Ideally, this progress should include involving potential trial participants in instrument development and evaluation.

### Strengths and limitations

This study included a detailed systematic search to identify patient reported measures of informed consent to clinical trials and included rigorous methods to identify and code relevant domains across included patient reported measures. Searching was applied across a range of databases and incorporated search filters designed to ensure wide coverage and capture of relevant measurement tools. It included studies from a variety of trial contexts and geographical settings which maximises the relevance of review to different settings. It does, however, have some weaknesses. The coding of items was conducted by two authors independently with a third author checking a random sub-sample. The coding was informed by directed content analysis, which has some level of interpretation required during the analysis. Therefore, whilst a systematic and rigorous approach, like many qualitatively interpretive approaches it is subjective and it is possible that if conducted by other researchers (with different perspectives and lenses) that a different overall result may emerge. No formal assessment of inter-rater reliability was conducted, however, informal assessments of consistency did not highlight any major problems. In addition, we did not conduct any formal assessment of the methodological quality of instruments identified in included studies. Whilst this was not the aim of the study it could have provided a conclusion about which (if any) of the existing measures is the most methodologically sound.

## Conclusions

This study has demonstrated the variability in the theoretical underpinning, development and domain coverage of existing patient-reported measures of informed consent for clinical trials. The dominant focus of tools to date has been limited to measuring understanding of items deemed important by researchers—the conceptualisation of informed consent could benefit from being extended to include broader considerations of decision-making. Meaningful involvement of potential trial participants during development of measures ‘critical for tool relevance’ is also lacking. The findings from this work provide evidence to support efforts to identify the key domains (of relevance to all stakeholders) which could be measured to assess the adequacy of the existing (and efforts to improve) informed consent process for clinical trials.

## Supporting information

S1 Appendix Search strategies for Medline, Embase, CINAHL Land PsychINFO. (PDF)

S1 Table Characteristics of included studies. (PDF)

S1 Text Coding of items across instruments. (XLSX)
