# Supplementary material for: Patient reported measures of informed consent for clinical trials: A systematic review
Source: PLoS One. 2018 Jun 27;13(6):e0199775. doi: 10.1371/journal.pone.0199775 (PMC6021104; doi:10.1371/journal.pone.0199775)
Supplement: S1 Appendix — (PDF) [file pone.0199775.s001.pdf]

S1. Appendix: Search strategies

MEDLINE & EMBASE

1 informed consent/
2 (informed adj3 (consent or decision? or choice)).tw.
3 (consent$ adj2 (process or form$ or document$ or written or verbal)).tw.
4 informed consent.kw.
5 or/1-4
6 exp research subjects/
7 patient participation/
8 ((participat$ or tak$ part or enrol$ or recruit$) adj7 (research or stud$ or experiment$ or trial?)).tw.
9 refusal to participate/
10 decision making/
11 exp "clinical trials as topic"/
12 (research subjects or patient participation).kw.
13 or/6-12
14 "surveys and questionnaires"/
15 interview/
16 exp "Outcome Assessment (Health Care)"/
17 Health Care Quality/
18 evaluation studies/
19 comprehension/
20 (evaluation or questionnaire or interview).kw.
21 therapeutic misconception/
22 understanding.tw.
23 (consent adj4 (scal$ or instrument$ or measure$ or tool$ or score$ or evaluat$)).tw.
24 or/14-23
25 5 and 13 and 24
26 instrumentation.fs.
27 validation studies/
28 "reproducibility of results"/
29 reproducib$.tw.
30 Psychometrics/
31 (psychometr$ or clinimetr$ or clinometr$).tw.
32 observer variation/
33 observer variation.tw.
34 discriminant analysis/
35 (reliab$ or valid$ or coefficient$ or "internal consistency").tw.
36 (cronbach$ and (alpha or alphas)).tw.
37 ("item correlation" or "item correlations" or "item selection" or "items selections" or "item reduction" or "item reductions").tw.
38 (agreement or precision or imprecision or "precise values" or test-retest).tw.
39 (test and retest).tw.
40 (reliab$ and (test or retest)).tw.
41 (stability or interrater or inter-rater or intrarater or intra rater).tw.
42 (intertester or inter-tester or intratester or intra-tester).tw.
43 (interobserver or inter-observer or intraobserver or intra-observer).tw.
44 (intertechnician or inter-technician or intratechnician or intra-technician).tw.
45 (interexaminer or inter-examiner or intraexaminer or intra-examiner).tw.
46 (interassay or inter-assay or intraassay or intra-assay).tw.

47 (interindividual or inter-individual or intraindividual or intra-individual).tw.
48 (interparticipant or inter-participant or intraparticipant or intra-participant).tw.
49 (kappa or kappa's or kappas).tw.
50 ("coefficient of variation" or repeatab$).tw.
51 ((replicab$ or repeated) and (measure or measures or findings or result or results or test or tests)).tw.
52 (generaliza$ or generalisa$ or concordance).tw.
53 (intraclass and correlation$).tw.
54 (discriminative or "known group" or "factor analysis" or "factor analyses" or " factor structure" or "factor structures").tw.
55 (dimensionality or subscale$ or "multitrait scaling analysis" or "multitrait scaling analyses").tw.
56 ("item discriminant" or "interscale correlation" or "interscale correlations").tw.
57 ((error or errors) and (measure$ or correlat$ or evaluat$ or accuracy or accurate or precision or mean)).tw.
58 ("individual variability" or "interval variability" or "rate variability" or "variability analysis").tw.
59 (uncertainty and measurement).tw.
60 (uncertainty and measuring).tw.
61 ("standard error of measurement" or sensitiv$ or responsive$).tw.
62 (limit and detection).tw.
63 "minimal detectable concentration".tw.
64 interpretab$.tw.
65 (small$ and (real or detectable) and (change or difference)).tw.
66 ("meaningful change" or "minimal important change" or "minimal important difference" or "minimally important change" or "mniimally important difference").tw.
67 ("minimal detectable difference" or "minimal detectable change" or "minimally detectable change" or "minimally detectable difference").tw.
68 ("minimal real change" or "minimal real difference" or "minimally real change" or "minimally real difference").tw.
69 ("ceiling effect" or "floor effect" or "item response model" or irt or rasch).tw.
70 ("differential item functioning" or dif or "computer adaprive testing" or "item bank" or "cross-culture equivalence").tw.
71 or/26-70
72 5 and 13 and 71
73 72 not 25

CINAHL

17	S9 AND S16
S16	S10 OR S11 OR S12 OR S13 OR S14 OR S15
S15	TX understanding OR TX ((consent N4 (scal* or instrument* or measure* or tool* or score* or evaluat*)))
S14	(MH "Evaluation Research")
S13	(MH "Quality of Health Care")
S12	(MH "Outcome Assessment")
S11	(MH "Interviews+")
S10	(MH "Questionnaires+")
S9	S1 OR S8

S8 S2 AND S7

S7 S3 OR S4 OR S5 OR S6

S6 (MH "Consumer Participation")

S5 (MH "Refusal to Participate")

S4 (MH "Research Subject Recruitment")

S3 TX ((participat* or tak* part or enrol* or recruit*) N7 (research or stud* or experiment* or trial*))

S2 TX ((informed N3 (consent or decision* or choice))) OR TX ((consent* N2 (process or form* or document* or written or verbal)))

S1 (MH "Consent (Research)")

PsychINFO

1 informed consent/
2 (informed adj3 (consent or decision* or choice)).tw.
3 (consent$ adj2 (process or form$ or document$ or written or verbal)).tw.
4 or/1-3
5 client participation/
6 experimental subjects/
7 decision making/
8 ((participat$ or tak$ part or enrol$ or recruit$) adj7 (research or stud$ or experiment$ or trial?)).tw.
9 or/5-8
10 questionnaires/ or measurement/
11 interviews/
12 comprehension/
13 evaluation/
14 understanding.tw.
15 ((participat$ or tak$ part or enrol$ or recruit$) adj7 (research or stud$ or experiment$ or trial?)).tw.
16 or/10-15
17 4 and 9 and 16
18 (2016$ or 2015$ or 2014$).up.
19 17 and 18
