# Supplementary material for: Patient reported measures of informed consent for clinical trials: A systematic review
Source: PLoS One. 2018 Jun 27;13(6):e0199775. doi: 10.1371/journal.pone.0199775 (PMC6021104; doi:10.1371/journal.pone.0199775)
Supplement: S1 Table — (PDF) [file pone.0199775.s002.pdf]

S1 Table. Characteristics of included studies.

	Author	Year	Country	Population	Sample size (n)	Response rate (n)	Age, mean (SD)	Female (%)	Ethnicity	Employment status (%)	Education status
1	Appelbaum *et al*	1999	USA	Clinical trial participants in a psychotherapy RCT for recurrent depression.	26	26	39 (10.45, range24-49)	100	No data	No data	No data
2	Appelbaum *et al*	2012	USA	Clinical trial participants from 4 academic medical centres in different geographic regions. Various trial phases and clinical areas.	220	189 (86%)	No data	45	No data	Employed 55<br>Retired 40<br>Looking for work 14<br>Domestic duties 5<br>Other 40	No data
3	Chou & O'Rourke	2012	Canada<br>USA<br>Australia<br>New Zealand	Study 1: Instrument development. Community dwelling mid-life and older adults recruited through membership lists, websites, and postings on older adult bulletin boards. Hypothetical clinical trial example.<br><br>Study 2: Instrument validation. Clinical trial participants	Study 1 464<br><br>Study 2 37	No data	Study 1<br>59.8 (6.2, range 50-84)	Study 1 59	Study 1<br>White/ Caucasian/ European 94.5%	Study 1<br>Retired 54<br>Full-time 27<br>Part-time 13	Study 1<br>Average 13.9 years formal education (SD=4, range 0-25)
4	Guarino *et al*	2006	USA	Set within an RCT of Gulf War Veterans' illnesses.	1086	3 months: 906(83%)<br>6 months :929 (86%)<br>12 months: 910 (84%)	40.7 (range 27-72)	15	White non-hispanic 53%<br><br>Black non-hispanic 24%<br>Hispanic 20%	No data	Mean education 14.1 yrs (SD=1.9)
5	Hutchison *et al*	2007	UK	Hospital based. Various participant groups: research nurses from a variety of specialties; and cancer patients (2 groups: previous RCT and no previous RCT)	78	78<br>Study recruited additional participants to account for non-responders	Patient: Previous RCT 57 (range 38-76)<br>Patient: No RCT 63 (range 43-81)<br><br>Research Nurse 41 (range 28-57)	Patient: Previous RCT 62<br>Patient: No RCT 70<br><br>Research Nurse 92	No data	No data	No data
6	Jeste *et al*	2007	USA				Patients	Patients 36	Patient Healthy	No data	No data

				Set within a larger study of informed consent among middle aged and older persons with schizophrenia. Simulated clinical drug trial.	127 patients<br>30 health comparators	127 patients<br>30 health comparators	51.8 (7.3)<br>Healthy comparators 55.9 (9.0)	Healthy comparators 57	White 63 73<br>Black 17 10<br>Latino 11 7<br>Other 9 10		
7	Joffe *et al*	2001	USA	Patients and parents of paediatric patients enrolled in Phase I, II or III clinical trials. Tool was developed with intention to be used across clinical areas.	Pilot 1: 9 (5 patients and 4 parents)<br><br>Pilot 2: 10 (7 patients and 3 parents)	Not reported	No data	No data	No data	No data	Pilot 1: not reported<br><br>Pilot 2: ranged from high school to graduate.
8	Miller C *et al*	1996	USA	Set within 4 prospective double-blind RCTs of anti-infective agents.	275	275 Reliability measured on 50 and validity on 200 responses.	36 (12.8)	63	No data	No data	Mean yrs 14.4 (SD = 2.3, range 10-24)
9	Miller J *et al*	2011	USA	Online survey populations. The probability samples were weighted to reflect existing Population Survey findings.	Sample 1 (public) : 1200<br><br>Sample 2 (cancer survivor):2057	Sample 1 (public) : 1027<br><br>Sample 2 (cancer survivor):1788	Sample / 1 (n) / 2 (n)<br>18-24 / 91 / 34<br>25-34 / 210 / -<br>35-44 / 234 / 117<br>45-54 / 197 / 223<br>55-64 / 132 / 331	Sample 1 (public) : 52<br><br>Sample 2 (cancer survivor): 54	No data	No data	Sample / 1 (n) / 2 (n)<br>< high school / 131 / 82<br>High school / 367 / 386<br>Some college / 283 / 645<br>Baccalaureate / 145 / 393<br>Graduate or professional / 101 / 280

							65-74 109 467<br>>75 54 614							
10	Miller V *et al*	2011	USA	Parents/carers, of seriously ill children, recruited through paediatric hospital and who had made decision in the past days either about research (62.1%) or informed consent for treatment (32.9%).	250	231 (219 included in analysis)	37.1 (8.1)	74	Black Or African American	46	Family income ($)		Some high school	12
									American Indian/Alaskan Native	1	<19999	26	High school	32
									Asian	10	20000-39999	39	Vocational or some college	72
									White	147	40000-59000	35	College degree	56
									Other	14	60000-79000	40	Postgrad	12
									Missing	1	80000-99999	27	Professional or graduate degree	34
											>100000	49		
											Missing	3		
11	Porteri C *et al*	2008	Italy	Set within 2 Phase III placebo controlled drug RCTs for Alzheimer's disease.	42 patients<br>21 caregivers	42 patients<br>21 caregivers	Patients 74.6 (7.3, range 60-88)<br>Caregivers 53.7 (11.7, range 35-76)	Patients 71<br>Caregivers 62	No data		No data		No data	
12	Prentice *et al*	2007	USA	Schizophrenia patients enrolled in clinical trials	11	11	No data	No data	No data		No data		No data	
13	Resnick *et al*	2007	USA	Nursing home residents being recruited for to an RCT of a restorative care intervention.	346	346	86.1 (7.3)	84	95% Caucasian		No data		No data	
14	Sugarman *et al*	2005	USA	Set within 8 different parent RCTs	632	632	67 (7.2)	26	93% white<br>4% black<br>3% other		No data		72% had some college education	
